# Inter-observer Variability in Histomorphological Evaluation of Non-neoplastic Liver Biopsy Tissue and Impact of Clinical Information on Final Diagnosis in Shahid Beheshti University of Medical Sciences Affiliated Hospitals

**DOI:** 10.30699/ijp.2019.99566.1985

**Published:** 2019-08-01

**Authors:** Zeinab Kishanifarahani, Mahsa Ahadi, Behrang Kazeminejad, Tahmineh Mollasharifi, Malihe Saber Afsharian, Amir Sadeghi, Farahnaz Bidari, Elena Jamali, Niki Hasanzadeh, Abolfazl Movafagh, Arash Dehghan, Arsham Moradi, Afshin Moradi

**Affiliations:** 1 Cancer Research Center, Shohada-e-Tajrish Educational Hospital, Faculty of Medicine, Shahid Beheshti University of Medical Science, Tehran, Iran; 2 Pathology Department, Azad Medical University, Mashhad, Iran; 3 Research Center for Gastroenterology and Liver Diseases, Taleghani Educational Hospital, Faculty of Medicine, Shahid Beheshti University of Medical Science, Tehran, Iran; 4 Pathology Department, Hamadan University of Medical Sciences, Hamadan, Iran; 5 University of Toronto, Department of Biology, Toronto, Canada

**Keywords:** Liver biopsy, Pathology, Inter-observer, Grading, Staging

## Abstract

**Background & Objective:**

Liver biopsy is the main method for grading and staging liver disorders, but the effects of clinical information and optimal biopsy specimen size on interpretation remain contentious. The aim of the study was to evaluate the impact of clinical information and quality of liver specimen on inter-observer agreement for liver disease.

**Methods:**

A total of 289 consecutive biopsy specimens from 2010 to 2017 were re-evaluated by five pathologists using the modified Ishak and non-alcoholic fatty liver diseases (NAFLD) activity score (NAS) systems. Detailed clinical information was extracted from medical records of patients and the size of all liver biopsy samples was recorded.

**Results:**

Full agreement between primary diagnosis and final diagnosis was obtained in 214 cases (74%). The remaining cases, namely 22 (7.6%) and 53 (18.3%) biopsies had minor and major diagnostic discrepancies, respectively. The results showed that the overall agreement was significantly higher in cases with complete clinical information than patients without any clinical information and even with partial clinical information (*P*<0.001). Interestingly, no significant difference in inter-observer agreement was achieved with a length over 20 mm (*P*=0.181). However, the inter-observer variation significantly decreased when the number of portal tract was more than 10 (*P*=0.001).

**Conclusion:**

This study identified the impact of clinical information and the number of portal tracts as the key factors to diagnosis. Therefore, request forms for liver biopsies should always be accompanied with the clinical history. Moreover, adequacy of biopsy specimens is very useful for accurate evaluation of samples by pathologists.

## Introduction

Liver biopsy is an investigation tool that is widely used for staging and grading of diffuse liver disease and subsequent management and follow-up of patients with chronic liver conditions, despite the rise of non-invasive methods (1-4). Liver biopsy provides clues about fibrosis, inflammation, steatosis, necrosis, cirrhosis, and other histomorphological findings with prognostic and predictive potential (5). It is identified as a benchmark for the diagnosis and evaluation of fibrosis extent in chronic liver diseases (6). Evaluation of pathologic findings provides more tips that could be helpful for clinical care, including grading the severity of the diseases (severity of inflammation in chronic autoimmune or viral hepatitis and extent in fatty liver disease) and staging of fibrosis in chronic viral, autoimmune hepatitis, and hepatic steatosis (7-9). Furthermore, liver biopsy may unveil abnormalities such as iron overload and α1-antitrypsin globules not detectable by other methods such as imaging and laboratory tests (9).

The purpose of biopsy is to achieve objective data about the condition of the liver tissue (10). However, there are several elements that influence the objectivity of this investigation (11, 12). The pathologist’s expertise is one of the most crucial factors impacting the inter-observer agreement on liver biopsy analysis. Mistakes in the interpretation of liver biopsy by general pathologists have been reported about 25% in previous studies (13, 14). Many studies have shown discrepancies of opinion between pathologists when a second review is conducted (13, 15). Therefore, use of second opinions from expert liver pathologists is recommended (13, 16). Sufficient clinical information such as patient demographic characteristics, indication of liver biopsy, serologic test results, and image-guided liver biopsy are other effective factors for better diagnosis and decision-making processes (17). In order to reduce the risk of error in the commentary of liver biopsy, it is mandatory that the volume of the biopsy specimen be adequate. Reliable analysis of liver biopsy depends on the dimension of the biopsy sample in terms of the length and number of portal tracts (13, 18, 19). According to the guidelines presented by the Royal College of Pathologists (RCPath), the ideal biopsy sample for evaluating the scheme of injury, grading inflammation, and staging fibrosis is 20 mm or more in length and/or contains more than 10 whole portal spaces (20-22). Nevertheless, the effect of sufficient clinical information and optimal size of biopsy specimen on the diagnosis remains an open field and controversial among liver pathologists. 

The objectives of this cross-sectional study were as follows: evaluating the morphological findings of consecutive percutaneous needle liver biopsy samples, assessing the sufficient clinical information and the adequacy of liver biopsy specimens, investigating the inter-observer agreement between primary and final diagnosis; and determining the impact of clinical information and quality of liver specimen on inter-observer agreement.

## Materials and Methods

This cross-sectional study included a total of 298 consecutive percutaneous needle liver biopsies of adult patients with liver dysfunction which were biopsied in 4 different hospitals (Shohada-e-Tajrish, Taleghani, Shahid-Modarres, and Loghman Hakim) in Tehran, Iran between 2010 and 2017. Patients with a history of liver transplant and those younger than 18 were excluded from the study. All liver biopsy specimens were re-evaluated by five pathologists of Shahid Beheshti University (SBU), Tehran, Iran, with appropriate experience to score chronic liver disease stage. All pathologists were blinded to the results of the primary diagnosis and identity of the first evaluator. The consensus meetings were organized and modified Ishak scoring system with NAFLD activity score (NAS) for liver biopsy assessment was chosen for the study, both of which are globally accepted; in addition, the initial diagnosis was based on these criteria (23, 24). The liver fibrosis and necroinflammatory activity of chronic hepatitis were assessed using the modified Ishak scoring system (24). Grading and staging of NAFLD and non-alcoholic steatohepatitis (NASH) were assessed according to the NAFLD activity score (NAS) of the Pathology Committee of the NASH Clinical Research Network; furthermore, the histologic features of NAS were based on a combination of features including steatosis, hepatocyte ballooning, and lobular inflammation (23, 25).

All slides were re-evaluated by a panel of pathologists at the consensus meeting and the recorded results were considered as the final diagnosis. Then, the inter-observer agreement between primary diagnosis and final diagnosis was assessed. After comparison, the results were placed in one of the following three categories: full agreement, minor discrepancy, and major discrepancy. Full agreement was defined as: “final diagnosis is identical to the previous diagnosis”; by minor discrepancy we meant: “the second diagnosis is not completely different from the initial diagnosis defined in such a way that treatment is significantly affected”; and the major discrepancy referred to the idea that: “the second diagnosis is completely different from the initial diagnosis”.

For the purpose of the study, the sufficiency of clinical information and the adequacy of liver biopsy specimen were recorded for all cases. The patient demographic characteristics (sex and age), previous pathologic reports, the clinical information and the laboratory data (liver function tests and serological studies) were collected from the patient chart. Adequacy of clinical information was categorized into three groups:

**Group 1:** those without any clinical information; 

**Group 2:** those with partial clinical information; and

**Group 3:** those with complete clinical information. 

In addition, liver biopsy core size and the number of portal tracts were classified into three groups of adequate (20 millimeter (mm) or more in length and containing 10 or more portal tracts), compromised (under 20 mm in length and containing 6–10 portal tracts), and inadequate (containing fewer than 6 portal tracts) (26). Then, the impact of clinical information and quality of liver specimen on inter-observer agreement for liver diseases was evaluated. 

The collected data were analyzed with statistical package for the social sciences (SPSS) software (version 21.0). Also, Chi-square test or Fisher’s exact test were performed for categorical variables.

## Results

Patient Characteristics and Clinical Information

A total of 289 biopsies of adult cases were evaluated. The mean age of participants was 46.6 ± 0.9 years and the range was 18 to 86 years. Out of 289 participants, 150 (51.9%) were male. The mean age of male and female was 46.9±16.7 and 46.4±14.7 years, respectively. After the reassessment of 289 liver biopsies by a group of pathologists and comparing them with primary diagnosis, full agreement was obtained in 214 cases (74%); meanwhile, 22 (7.6%) and 53 (18.3%) samples had minor and major diagnostic discrepancy, respectively. All patient characteristics, clinical information, liver biopsy core length, number of portal tracts, and the inter-observer agreement between primary and final diagnosis are presented in Table 1.

**Table 1:** Patient characteristics, clinical information, adequacy of liver biopsy and the percentage of the agreement between primary and final diagnosis

Variables		Frequency (%)
Sex	Male Female	150 (51.9) 139 (48.1)
Age Groups	≤ 40 40-60 ≥ 60	122 (42.2) 108 (37.4) 59 (20.4)
Clinical Information sufficiency*	Group 1 Group 2 Group 3	9 (3.1) 218 (75.4) 62 (21.5)
Core length	< 10 mm 10-20 mm >20 mm	16 (5.5) 105 (36.3) 168 (58.1)
Number of portal tracts	<6 6-10 >10	81(28) 137 (47.4) 71 (24.6)
Inter-observer agreement	Full agreement Minor discrepancy Major discrepancy	214 (74) 22 (7.6) 53 (18.3)

* Group 1: without any clinical information, Group 2: with partial clinical information, Group 3: with complete clinical information

Impact of Clinical Information and Adequacy of Liver Biopsy on Inter-observer Agreement

Table 2 shows the impact of clinical information and adequacy of liver biopsy based on core length and the number of portal tracts of specimens on inter-observer agreement between primary and final diagnosis. The results show that the overall agreement was significantly higher in cases with complete clinical information than patients without any clinical information and even with partial clinical information (80.6% vs. 22.2%, *P*<0.001). Interestingly, no significant difference in inter-observer agreement was achieved with a length over 20 mm compared to those less than 20 mm (78% vs. 68.6%, *P*=0.181). However, inter-observer variation significantly decreased when the number of portal tract was more than 10 compared to less than 10 (5.6% vs. 32.6%, *P*=0.001).

**Table 2:** Clinical information and adequacy of liver biopsy samples according to the agreement between primary and final diagnosis

Variables		Agreement			Total	P-value
		Full agreement	Minor discrepancy	Major discrepancy		
Clinical information sufficiency	Group 1 Group 2 Group 3	2 (22.2) 162 (74.3) 50 (80.6)	2 (22.2) 15 (6.9) 5 (8.1)	5 (55.6) 41 (18.8) 7 (11.3)	9 (100) 218 (100) 62 (100)	<0.001*
Core length	< 20 mm ≥ 20 mm	83 (68.6) 131 (78)	12 (9.9) 10 (6)	26 (21.5) 27 (16.1)	121 (100) 168 (100)	0.181
Number of portal tracts	< 10 ≥ 10	147 (67.4) 67 (94.3)	21 (9.6) 1 (1.4)	50 (23) 3 (4.2)	218 (100) 71 (100)	0.001*
Total		214 (74)	22 (7.6)	53 (18.3)	289 (100)	

* Group 1: without any clinical information, Group 2: with partial clinical information, Group 3: with complete clinical information, P-value <0.05 (statistically significant)

Inter-observer variations

In this study, 75 (26%) samples of liver biopsy had discrepancy when the second review was implemented. Table 3 represents the minor and major discrepancy interpretations encountered and their related frequency. The results indicated that: 15 (28.3%) cases of the major interpretation errors were related to the NASH process, in 12 (22.6%) cases chronic hepatitis was overlooked in the initial diagnosis, 10 (18.9%) cases were related to determining the presence or absence of advanced liver disease (cirrhosis), 9 (17%) cases were on patients with chronic cholestasis disorders, 4 (7.5%) cases with hepatocellular process, and 3 (5.6%) cases were inadequate for diagnosis In the minor discrepancy group, 13 (59.1%) cases belonged to the normal specimens which were overdiagnosed as mild portal inflammation in the first pathology reports. Also, in this group there were seven (31.8%) cases with active hepatitis that were diagnosed as chronic hepatitis in re-evaluation, and two (9.1%) cases with NAFLD that were reported as mild steatosis in primary diagnosis.

**Table 3:** Major and minor discrepancy interpretations encountered and their frequency (n=75)

Discrepancies	Primary Diagnosis	Final Diagnosis	Frequency (%)
Minor	Mild Portal Inflammation	Normal	13/22 (59.1)
	Active Hepatitis	Chronic Hepatitis	7/22 (31.8)
	Mild Steatosis	Moderate Steatosis	2/22 (9.1)
Major	No cirrhosis	Cirrhosis	4/53 (7.5)
	Cirrhosis	No cirrhosis	6/53 (11.4)
	Steatosis	NASH	9/53 (17)
Major	Chronic Hepatitis	Cholestasis disorders	9/53 (17)
		Normal	12/53 (22.6)
		NASH	6/53 (11.4)
		Solid mass	4/53 (7.5)
		Inadequate for diagnosis	3/53 (5.6)

Morphological and Histopathiological Findings

The three most frequent morphological changes seen in this study are inflammatory lesions, fibrosis, and steatosis with the frequencies of 56.1%, 26.6%. and 23.9%, respectively. Other morphological findings are presented in table 4. Fibrosis staging and necro-inflammatory activity of chronic hepatitis were assessed based on modified Ishak scoring system and the results are presented in table 5. The assessment of Histology Activity Index (HAI) showed minimal, mild, moderate, and severe chronic hepatitis in 29 (26.4%), 59 (53.6%), 17 (15.5%), and 5 (4.5%) patients, respectively. Fibrosis staging in 110 cases with chronic hepatitis reported that 27 (24.5%) patients, 32 (29.1%) patients, 8 (7.3%) patients, 13 (11.8%) patients, 5 (4.5%) patients, 18 (16.4%) patients, and 7 (6.4%) patients had 0 to 6 stages, respectively. NAFLD activity score (NAS) was used to evaluate steatohepatitis and steatofibrosis, the results of which are presented in table 6. NAFLD activity score based on steatosis, lobular inflammation, and hepatocyte ballooning identified 6 (27.3%), 9 (40.9%), and 7 (31.8%) patients as mild, moderate, and severe grade of NAFLD. Also, 12 (54.5%), 5 (22.7%), 1 (4.5%), 3 (13.6%), and 1 (4.5%) participants had 0, 1A, 1C, 2, and 3 stages of steatofibrosis, respectively. 

**Table 4:** Morphological findings of lesions by liver biopsy

Final Diagnosis		Frequency (%)	Total
Inflammatory Lesions	Portal Inflammation	152/162 (94)	162 (56.1)
	Lobular Inflammation	8/162 (5)	
	Mix of portal and lobular inflammation	2/162 (1)	
Cholestasis disorders			34 (11.8)
Hepatic Mass	Primary Solid Mass	7/62 (11.3)	62 (21.4)
	Metastatic Solid Mass	18/62 (29)	
	Primary or Metastatic Solid mass	37/62 (59.7)	
Bile Ductular Reaction	Acute Ductular Reaction	14/47 (29.8)	47 (16.3)
	Chronic Ductular Reaction	33/47 (70.2)	
Steatosis			67 (23.9)
Steatohepatitis	Mild	6/22 (27.3)	22 (7.6)
	Moderate	9/22 (40.9)	
	Severe	7/22 (31.8)	
Fibrosis			77 (26.6)

**Table 5:** Results of HAI grading and fibrosis staging based on Ishak scoring system

HAI Findings	Score	Frequency (%)
Absent	0	0
Minimal	1	2 (1.8)
	2	12 (10.9)
	3	15 (13.6)
	1-3	29 (26.4)
Mild	4	20 (18.2)
	5	10 (9.1)
	6	8 (7.3)
	7	12 (10.9)
	8	9 (8.2)
	4-8	59 (53.6)
Moderate	9	7 (6.4)
	10	6 (5.4)
	11	2 (1.8)
	12	2 (1.8)
	9-12	17 (14.8)
Severe	13	3 (2.7)
	14	1 (0.9)
	15	1 (0.9)
	16	0
	17	0
	18	0
	13-18	5 (4.5)
Maximum possible score for grading	18	110 (100)
Fibrosis stage	**Score**	**Frequency (%)**
No fibrosis	0	27 (24.5)
Fibrous expansion of some portal areas, with or without short fibrous septa	1	32 (29.1)
Fibrous expansion of most portal areas, with or without short fibrous septa	2	8 (7.3)
Fibrous expansion of most portal areas with occasional portal to portal (P-P) bridging	3	13 (11.8)
Fibrous expansion of portal areas with marked bridging (P-P) as well as portal-central (P-C)	4	5 (4.5)
Marked bridging (P-P and/or P-C) with occasional nodules (incomplete cirrhosis)	5	18 (16.4)
Cirrhosis	6	7 (6.4)
Maximum possible score for staging	6	110 (100)

**Table 6:** Results of NAFLD activity grading and staging system

NAFLD activity	Score	Frequency (%)
Mild	0	0
	1	0
	2	6 (27.3)
	0-2	6 (27.3)
Moderate	3	3 (13.6)
	4	6 (27.3)
	3-4	9 (40.9)
Severe	5	5 (22.7)
	6	2 (9.1)
	7	0
	8	0
	5-8	7 (31.8)
Maximum possible score for grading	**0-8**	**22 (100)**
Steatofibrosis stage	**Score**	**Frequency (%)**
No fibrosis	0	12 (54.5)
Perisinusoidal or periportal	1	0
Mild, zone 3, perisinusoidal	1A	5 (22.7)
Moderate, zone 3, perisinusoidal	1B	0
Portal/periportal only	1C	1 (4.5)
Both perisinusoidal and portal/periportal	2	3 (13.6)
Bridging fibrosis	3	1 (4.5)
Cirrhosis	4	0
Maximum possible score for Staging	**4**	**22 (100)**

## Discussion

Most of non-neoplastic hepatic disorders have overlapping histomorphologic features and core needle biopsy specimens do not usually contain specific features to allow absolute diagnosis. In the absence of necessary laboratory, imaging, and clinical data, pathologists may be able to make a pattern diagnosis (9, 27). For instance, alcoholic and non-alcoholic fatty liver diseases have practically the same morphologic patterns and differentiation between these diseases mandates additional clinical detail (28). Furthermore, differential diagnosis of viral hepatitis and autoimmune hepatitis (AIH) without clinical information is very difficult due to similar histopathological features, while the distinction between them is very critical, because therapeutic strategies are different for each one. Viral hepatitis is commonly treated with alpha-interferon, which can promote auto-immune responses in the liver. In contrast, autoimmune hepatitis is treated with immunosuppressive drugs that can further multiply the virus in viral hepatitis cases (29, 30). 

The results showed that overall agreement was significantly higher in cases with complete clinical information than patients without any clinical information and even with partial clinical information (80.6% vs. 22.2%, *P*<0.001). Therefore, the availability of clinical information is one of the factors affecting the observational agreement between the primary diagnosis and the ultimate diagnosis in these patients. Based on the results obtained in this study, it is recommended that the request forms for liver biopsy be accompanied with the patients’ clinical history, including the results of any relevant laboratory and imaging investigations. 

The liver tissue specimen must be adequate in size to reduce interpretation flaw and intra-observer discrepancies. In this study, no significant difference in the inter-observer agreement was achieved with a length over 20 mm and less than 20 mm (78% vs. 68.6%, *P*=0.181). However, inter-observer variation significantly decreased when the number of portal tract was more than 10 (32.6 vs. 5.6, *P*=0.001). The ideal size of liver biopsy for histological analysis is still an open field among liver pathologists. Colloredo *et al.* (31) demonstrated that a sample greater than or corresponding to 20 mm in length and at a minimum 11 portal tracts was required for correct evaluation. Bedossa *et al.* (32) found that 25 mm in length was necessary to precisely stage fibrosis in hepatitis C according to the METAVIR system. Conversely, Schiano *et al.* (33) did not find a substantial difference in fibrosis stage when evaluating various volumes of the same liver specimen. These differences can be attributed to several reasons, including the clinician's experience (who did the biopsy) and the kind of needle used for biopsy. Previous studies have found a significant relation between numbers of adequate cores and experience of biopsy taker (18, 34, 35). In addition, some studies have shown the correlation of liver biopsy adequacy with the types of needle used (10, 36, 37). 

Numerous studies have shown the discrepancies of opinion among pathologists when asked for a second review (13, 15). Some studies showed the inter- and intra-observer variability in grading and staging of chronic hepatitis (38, 39), or non-alcoholic liver steatosis (40) in a second review. In non-alcoholic fatty liver, Younossi *et al.* (41) found that criteria of inflammation were not as reliable as standards of fibrosis. Some situations demonstrated 96% full agreement (42), whereas Theodossi *et al.* (43) reported only 15% agreement among six pathologists in examining 60 liver samples. In the current study, 74% full agreement was obtained between primary and final diagnosis. We also found that 18.3% with major discrepancies had the potential to lead to inappropriate patient care. In addition, a major discrepancy between pathologists was reported 28% by Bejarano *et al.* in 2001 (13). However, further researche is needed to clear up the impact of clinical treatment and profitability of re-evaluation of liver biopsy specimens.

 The histologic findings are categorized into ballooning of hepatocytes, steatosis, cholestasis, necrosis and/or apoptosis, inflammation, regenerative changes, and architecture alteration with or without fibrosis (7). The mentioned findings, presented individually or simultaneously, create patterns of liver damage which contain several differential diagnoses. The three most common morphological changes seen in this study are inflammatory lesions, fibrosis, and steatosis. Inflammatory lesions were the most common findings, which were observed in a wide variety of liver diseases that mainly include necro-inflammatory diseases, such as viral hepatitis, autoimmune, and drug induced hepatitis (44, 45). Similar to our study, in many studies inflammatory lesion of the liver was the most common morphological changes in the liver disease (8, 46, 47). In this study, inflammatory pattern of the liver accounted for 56.1% of cases with 94%, 5%, and 1% of portal inflammation, lobular inflammation, and mixture of portal and lobular inflammation, respectively. The type of inflammatory cells is important because it can provide a clue to a specific liver disease (7). In the current study, portal inflammatory cells were lymphocytic in 119 (78.3%) cases, mixed cells (lymphocytes, plasma cells, and eosinophil) in 29 (19.1%) cases, granulomatous inflammation in two (1.3%) cases, plasma cells in one (0.6%) case, and eosinophil cells in one (0.6%) case. The review of portal inflammatory activity in 151 cases showed mild, moderate, and severe inflammation in 77 (50.6%), 44 (28.9%), and 31 (20.5%) cases, respectively. On the other hand, assessment of lobular inflammatory cells in 8 cases showed non-sinusoidal pattern of lymphocytic, sinusoidal pattern of lymphocytic, and other patterns in 6 (75%), 1 (12.5%), and 1 (12.5%) cases, respectively.

The main strength of this study was the large number of liver biopsies that included a wide range of liver diseases. The accuracy of final diagnosis of liver biopsy specimens was high because it was based on the observations of the group of pathologists with appropriate experience on liver pathology. However, the study had some limitations. Since observations and findings of pathologists were not recorded as separate diagnosis, the inter-observer agreement between each five pathologists could not be calculated. 

## Conclusion

In conclusion, without having minimum clinical information and adequate liver biopsy samples, pathologists are not able to diagnose liver diseases correctly. Appropriate clinical data in terms of laboratory evaluations, imaging, and clinical findings can lead to a more accurate diagnosis. Based on our results, more precise diagnosis and evaluation was performed on the samples with more than 10 portal tracts and also those with complete clinical information or even with partial information than those without any clinical data. Request forms for liver pathology should always provide relevant clinical details. Clinicians make classification of morphologic changes more precise and reproducible by taking an adequate liver tissue sample.
